# Can intraocular pressure serve as a non-invasive surrogate marker for intracranial pressure following traumatic brain injury?

**DOI:** 10.1016/j.ccrj.2025.100161

**Published:** 2026-02-23

**Authors:** Bao N. Nguyen, Ella Stathis, Bang V. Bui, Lauren N. Ayton, David B. Grayden, Sam E. John, Janine Stubbs, Andrew Morokoff, Olivia Gigli, Brianna Tascone, Ryan Nolan, Emily J. See, Adam M. Deane, Yasmine Ali Abdelhamid

**Affiliations:** Department of Optometry and Vision Sciences, The University of Melbourne, Parkville, Victoria, Australia; Intensive Care Unit, The Royal Melbourne Hospital, Parkville, Victoria, Australia; Department of Critical Care, The University of Melbourne, Parkville, Victoria, Australia; Department of Optometry and Vision Sciences, The University of Melbourne, Parkville, Victoria, Australia; Department of Optometry and Vision Sciences, The University of Melbourne, Parkville, Victoria, Australia; Department of Surgery (Ophthalmology), The University of Melbourne, Parkville, Victoria, Australia; Centre for Eye Research Australia, East Melbourne, Victoria, Australia; Department of Biomedical Engineering, The University of Melbourne, Parkville, Victoria, Australia; Graeme Clark Institute for Biomedical Engineering, The University of Melbourne, Parkville, Victoria, Australia; Department of Biomedical Engineering, The University of Melbourne, Parkville, Victoria, Australia; Graeme Clark Institute for Biomedical Engineering, The University of Melbourne, Parkville, Victoria, Australia; Department of Medicine, The University of Melbourne, Parkville, Victoria, Australia; Statistical Consulting Centre, The University of Melbourne, Parkville, Victoria, Australia; Neurosurgery, The Royal Melbourne Hospital, Parkville, Victoria, Australia; Department of Surgery, The University of Melbourne, Parkville, Victoria, Australia; Intensive Care Unit, The Royal Melbourne Hospital, Parkville, Victoria, Australia; Intensive Care Unit, The Royal Melbourne Hospital, Parkville, Victoria, Australia; Department of Critical Care, The University of Melbourne, Parkville, Victoria, Australia

**Keywords:** Traumatic brain injury, Intracranial pressure, Intraocular pressure, Critical care

Raised intracranial pressure (ICP) is a life-threatening complication of traumatic brain injury (TBI) that requires urgent treatment. In the intensive care unit (ICU), ICP is monitored using invasive devices such as external ventricular drains or intraparenchymal monitors. While these devices are accurate, they can be challenging to insert and are associated with risks and complications. Moreover, outside the ICU, there may be settings where ICP monitoring is neither routine nor available (e.g. pre-hospital, emergency triage, low-income countries, or resource-constrained settings) where alternative cost-effective ways to monitor ICP are needed.

Intraocular pressure (IOP) has been proposed as a non-invasive surrogate marker for ICP^1^ since the eye and brain share adjoining fluid compartments, and IOP measurement can be safely performed at the bedside in ICU. While some studies show a positive correlation between ICP and IOP,[2], [3], [4], [5], [6], [7] with one meta-analysis reporting a pooled correlation coefficient of *r* = 0.44,^8^ other reports vary widely in their correlation measures.[9], [10], [11], [12], [13], [14], [15], [16], [17] However, prior work has been limited by the use of correlation analysis, heterogenous patient groups with diverse pathophysiology, and measurements of IOP and ICP collected at different times and in different body positions. In particular, head elevation—a possible confounder of the relationship between IOP and ICP—has never been controlled for previously.

In order to address these gaps, we conducted a prospective, single-centre, observational study in TBI patients undergoing invasive ICP monitoring. To determine if IOP can serve as a non-invasive surrogate marker for ICP, we conducted linear regression using a mixed model for repeated measurements in the same patient and explicitly accounted for head elevation.

Study procedures were conducted in the ICU of the Royal Melbourne Hospital, Australia, and approved as an evaluation activity (QA2022147) without requiring patient consent by the hospital’s Office for Research and Human Research Ethics Committee. Patients were included as part of a convenience sample if they were aged ≥18 years, consecutively admitted to the ICU with a TBI, and undergoing continuous ICP monitoring with either an external ventricular drain (EVD) or intraparenchymal monitor during the planned data collection period of 1 April 2023 to 30 November 2023. Patients were excluded if they had sustained facial/ocular trauma or had a history of intraocular surgery or ocular disease (e.g. glaucoma). Uninsured international patients were also excluded, in accordance with the local policy.

Demographic data and clinical characteristics appear in Table 1. Most patients (19, 95 %) were being monitored with an intraparenchymal ICP monitor. Patients were followed for up to 8 days or until study withdrawal due to discharge from the ICU, ICP monitor removal, or a positive COVID-19 test result due to infection control requirements. Standard intensive care management continued during the study period, including therapies for raised ICP. Four patients (20 %) had a decompressive craniectomy or craniotomy and evacuation of subdural haematoma prior to the commencement of the IOP data collection.

**Table 1:** Demographic, clinical, and study enrolment characteristics of the included study participants (N = 20).

Demographic and clinical characteristics	
Age, years, mean ± SD	45 ± 14
Sex, female (%)	2 (10 %)
Height, cm, mean ± SD	175 ± 8
Weight, kg, mean ± SD	84 ± 18
Body mass index, kg/m^2^, mean ± SD	28 ± 6
GCS score	
At scene of injury, median [IQR]	4 [3–8]
At hospital admission, median [IQR]	3 [3–7]
Pupil responsiveness at scene of injury, number (%)	
Both reactive	12 (60 %)
One reactive	3 (15 %)
None reactive	2 (10 %)
Not recorded	3 (15 %)
Pupil responsiveness at hospital admission, number (%)	
Both reactive	14 (70 %)
One reactive	2 (10 %)
None reactive	4 (20 %)
Not recorded	0 (0 %)
Pupil size at scene of injury, number (%)	
Both normal size	9 (45 %)
Both dilated	1 (5 %)
Both pinpoint	3 (15 %)
Anisocoric	1 (5 %)
Not recorded	6 (30 %)
Pupil size at hospital admission, mm	
Right eye, median [IQR]	3 [2–4]
Left eye, median [IQR]	3 [2–4]
Other injuries sustained, number (%)	
Face	7 (35 %)
Spine	7 (35 %)
Chest	11 (55 %)
Abdomen	7 (35 %)
Extremities	7 (35 %)
Neck	1 (5 %)
No other injuries	2 (10 %)
Source of ICU admission, number (%)	
Emergency department	15 (75 %)
Theatre (emergency)	5 (25 %)
Neurosurgical procedures performed prior to study enrolment, number (%)	4 (20 %)
Craniectomy with drainage of subdural haematoma	3 (15 %)
Craniotomy with drainage of subdural haematoma	1 (5 %)
ICP monitor type and brand, number (%)	
External ventricular drain	1 (5 %)
Pressio (Sophysa, Orsay, France)	1 (5 %)
Intraparenchymal	19 (95 %)
Pressio (Sophysa, Orsay, France)	8 (40 %)
Codman Cerelink (Integra LifeSciences, Mansfield, USA)	9 (45 %)
Brand not documented in medical record	2 (10 %)
APACHE III score, median [IQR]	62 [45–73]
Days of invasive mechanical ventilation, median [IQR]	9 [5–11]
Tracheostomy, number (%)	4 (20 %)
Renal replacement therapy, number (%)	2 (10 %)
Vasopressor use during ICU admission, number (%)	20 (100 %)
Additional therapies used for raised ICP during ICU admission^a^, number (%)	
Barbiturate coma	2 (10 %)
Muscle paralysis	12 (60 %)
Osmotic therapy	4 (20 %)
No therapies for raised ICP	7 (35 %)

Abbreviations: SD = standard deviation; IQR = interquartile range; GCS = Glasgow Coma Scale; ICU = Intensive Care Unit; ICP = intracranial pressure; APACHE = Acute Physiology and Chronic Health Evaluation.

a All patients received intravenous sedatives.

IOP was measured in both eyes (unless one eye showed ocular laceration, bruising or bleeding, orbital fracture, or significant eyelid swelling) by a trained ICU staff member using a handheld rebound tonometer with positional flexibility (IC200 model, iCare, Finland). ICU staff who were likely to be on call and available for patient testing during the study period (YAA, EJS, OG, BT, and RN) were trained by optometrists (BNN and LNA) on the tonometry technique, ensuring accurate positioning of the tonometer probe (i.e. perpendicular to the centre of the cornea at ∼5 mm working distance). Once correctly positioned, six consecutive measurements are automatically taken by the device and the mean IOP is displayed to 0.1 mmHg resolution. The device also provides an automated, proprietary reliability indicator based on the variation among six measurements. Only “reliable” measures (green reliability indicator) were included in the analysis as a quality control measure.

Data collection was subject to the patient’s care needs and staff availability. A specific protocol (e.g. of 2- or 4-hourly IOP measures) was not feasible in this context, given overriding patient care needs. Nevertheless, ICU staff aimed to measure IOP at three different times per day. All attempts, and reasons for an unsuccessful or missed IOP reading, were recorded wherever possible. Head elevation (degrees) and body position was recorded for each measurement. To ensure blinding, ICP at the time of IOP measurement was recorded by a second team member.

Patient study enrolment and data inclusion/exclusion are shown in Fig. S1 (Supplementary Material). No patients died during the study and no adverse events associated with IOP measurement were reported. The median time from ICU admission to study enrolment (i.e. first IOP measurement) was 13 h (interquartile range [IQR]: 9–24 h), and the median time from ICP monitor insertion to study enrolment was 14 h (IQR: 11–26 h). Over a median duration of 3 days of ICP monitoring (range: 1–8 days), 356 IOP measurements were attempted, of which 41 (12 %) were considered missed opportunities (e.g. patient in surgery, family visitation) and 19 (5 %) were determined to be unreliable by the tonometer. Patients were either lying on their side (lateral decubitus) or on their back at different degrees of head elevation (ranging from 0 to 55°). Only the 232 measurements while patients were lying on their back were analysed to ensure the two eyes were level, because of the complex interaction between body position, degree of head turn in the lateral decubitus position, and IOP.^18^^,^^19^

Characteristics of the ICP and IOP measurements included in the analysis are described in Table S1 (Supplementary Material). ICP ranged from −3 to 38 mmHg (mean ± SD: 12 ± 7 mmHg), while IOP ranged from 4 to 25 mmHg (10 ± 4 mmHg). The median number of simultaneous ICP and IOP measurements analysed per patient was 9 (range: 1–41). In 17 of 20 patients (85 %), there was a positive within-patient linear univariate association between ICP and IOP (Fig. 1). To estimate the association between IOP and ICP in the cohort, a mixed model with patient-specific intercepts accounted for repeated measures. Overall, the regression line represents that, for each 1 mmHg increase in IOP, ICP increased on average by 0.7 mmHg (95 % confidence interval: 0.5–0.9). The within-patient (residual) variance ($\sigma_{e}^{2})$ was 4.1; the between-patient variance ($\sigma_{u}^{2})$ was 42.25. This indicates that most of the variance in ICP comes from between-patient differences and IOP alone is insufficient to explain variation in ICP. Regression diagnostics appear in Fig. S2 and an overview of the models explored in the study analysis are described in Table S2 (Supplementary Material).

**Fig. 1:**
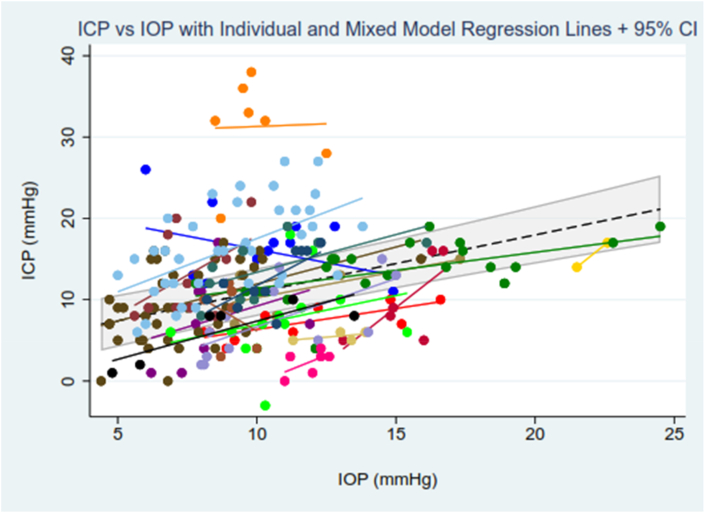
Individual regression lines depicting the relationship between ICP and IOP for each patient (except for one patient who contributed a single data point), and the overall linear relationship between ICP and IOP from the mixed model. Coloured symbols and associated lines represent each individual. The dashed line represents the most parsimonious linear model (described by ICP = 3.9 + 0.7 × IOP), with the 95 % confidence intervals of the regression line indicated by grey shading. Abbreviations: ICP = intracranial pressure; IOP = intraocular pressure; CI = confidence interval.

Differences in the observer taking the measurement (different ICU staff), and laterality of eye measurements (left vs right eye), did not influence the relationship between ICP and IOP (Table S3, Supplementary Material). The relationship between IOP and ICP was also unaffected by the degree of head elevation (Table S4, Supplementary Material). Although four ICP measurements were negative or zero, all available ICP data were incorporated in the analysis to reflect real-world variation. Sensitivity analysis with these values removed (2 % of all measurements) did not change the results (Table S5, Supplementary Material).

This study is the first to prospectively evaluate the relationship between ICP and IOP in TBI patients using modern hierarchical (mixed-effects) modelling to account for multiple measurements per patient. Previous work was limited by relying on correlation within heterogeneous patient populations and non-simultaneous measurements that could explain the large variability in correlation between studies.^8^ Here, we addressed several key limitations of previous reports by explicitly accounting for head elevation in a single body position and conducting simultaneous measurements and blinded assessments. Our results confirm a positive association between IOP and ICP at the population level as per previous meta-analysis results^8^ but IOP alone is insufficient to determine ICP. We found that degree of head elevation (a potential confounder overlooked previously) does not substantively change the relationship between ICP and IOP. This work has, for the first time, revealed previously unrecognised heterogeneity between individuals, which invites further studies to investigate potential contributing factors (other than head elevation) that may explain the variation in the IOP-ICP relationship.

The specific characteristics of our cohort are relevant to the interpretation of the data. In our cohort at a single hospital, we observed that TBI patients requiring an ICP monitoring device frequently exhibit ICP values within the normal range. Therefore, our data included a limited number of IOP measurements when ICP was greater than 20 mmHg, as raised ICP is rapidly treated in the ICU. While our work establishes the feasibility of characterising the IOP-ICP association at and above clinically relevant thresholds (20–22 mmHg) in similar clinical settings, future studies will require larger numbers of patients and readings to capture a greater range of ICP values.

In our patient cohort (a convenience sample of consecutive patients admitted to the ICU for invasive ICP monitoring), three people (15 %) had undergone a decompressive craniectomy and one patient (5 %) had drainage of a subdural haematoma via craniotomy, prior to enrolment in the study. In these contexts, the relationship between ICP and IOP may be different from that in patients without decompressive surgical procedures, as removing a portion of a patient’s skull could plausibly affect the pressure dynamics. However, a sub-analysis of these data is not feasible here as the numbers are too low to be informative. Similarly, given that only one patient had an EVD inserted, we cannot draw definitive conclusions about the differences in the relationship between IOP and ICP in patients with EVDs and those with intraparenchymal monitors. Nevertheless, we opted to include these cases as they reflect the real-world variation in TBI patients undergoing continuous ICP monitoring in the ICU, which was the primary inclusion criterion for our cohort of interest. While we did not collect measures of cerebral compliance to consider its potential interaction with IOP in this dataset, future studies could incorporate measures of ICP pulse wave P1:P2 ratio or compensatory reserve index (RAP) as dynamic, physiological markers of the brain’s ability to accommodate volume variations.

This work serves as a useful starting point for future studies aiming to define both the clinical threshold in absolute terms (change in IOP above a certain threshold) as well as enable IOP monitoring to assess trends over time. While our study demonstrates that IOP measurement in TBI patients is feasible, IOP alone cannot serve as a surrogate marker for ICP with accuracy, or over the range required, for ICU patient management. A rise in ICP from altered cerebrospinal fluid dynamics in the brain could theoretically transfer to the eye via the subarachnoid space of the retrobulbar region, which is continuous with that of the brain. The eye, however, has several compensatory mechanisms that can maintain IOP despite external pressures (like ICP), homeostatically balancing the inflow and outflow of fluid in the anterior chamber of the eye^20^ (e.g. aqueous humour production, episcleral venous pressure, flow resistance through trabecular meshwork), hence IOP may not always fully correlate with ICP. Other ophthalmic techniques such as ophthalmodynamometry (the measurement of blood pressure dynamics within retinal blood vessels)^21^ may give additional information, which, together with IOP, could provide more reliable non-invasive surrogates for ICP in the future. Given the limitations of IOP measurements, we are currently undertaking a program of work to develop a non-invasive portable device to approximate ICP using ophthalmodynamometry.

## Credit authorship contribution statement

BNN, BVB, LNA, DBG, SEJ, AM, AMD, and YAA contributed to the conception and design of the study. BNN, JS, ES, OG, BT, RN, EJS, BVB, and YAA contributed to acquisition and analysis of data. All authors contributed to drafting the text and/or preparing the figures.

## Data availability

The authors confirm that the data supporting the findings of this study are available within the article and its supplementary materials.

## Conflict of interest

The authors declare the following financial interests/personal relationships which may be considered as potential competing interests: Adam Deane reports financial support was provided by the National Health and Medical Research Council and the Victorian Medical Research Acceleration Fund. Lauren Ayton reports financial support was provided by the National Health and Medical Research Council and the Victorian Medical Research Acceleration Fund. Sam John reports financial support was provided by the Victorian Medical Research Acceleration Fund. David Grayden reports financial support was provided by the Victorian Medical Research Acceleration Fund. Bang Bui reports financial support was provided by the Victorian Medical Research Acceleration Fund. Andrew Morokoff reports financial support was provided by the Victorian Medical Research Acceleration Fund. Yasmine Ali Abdelhamid reports financial support was provided by the Victorian Medical Research Acceleration Fund. Andrew Morokoff reports a relationship with the Neurosurgical Society of Australasia that includes board membership. Lauren Ayton reports a relationship with Kiora Pharmaceuticals Inc that includes consulting or advisory, Janssen Pharmaceuticals Inc that includes consulting or advisory, Johnson & Johnson Vision that includes consulting or advisory, PYC Therapeutics that includes consulting or advisory, Novartis that includes speaking and lecture fees, Australian College of Optometry that includes board membership, and UsherKids Australia that includes board membership. Yasmine Ali Abdelhamid reports a relationship with Australian and New Zealand Intensive Care Society that includes board membership and declares that they are part of the CC&R editorial team as an associate editor. If there are other authors, they declare that they have no known competing financial interests or personal relationships that could have appeared to influence the work reported in this paper.
