# Supplementary material for: Can intraocular pressure serve as a non-invasive surrogate marker for intracranial pressure following traumatic brain injury?
Source: Crit Care Resusc. 2026 Feb 23;28(1):100161. doi: 10.1016/j.ccrj.2025.100161 (PMC12973525; doi:10.1016/j.ccrj.2025.100161)
Supplement: Multimedia component 1 [file mmc1.docx]

**SUPPLEMENTARY MATERIAL**

Can intraocular pressure serve as a non-invasive surrogate marker for intracranial pressure following traumatic brain injury?

**Table of contents**

[1. Supplementary figures 2](#_Toc210849621)

[2. Supplementary tables 4](#_Toc210849622)

[3. Supplementary methods 7](#_Toc210849623)

[3.1. Statistical analyses 7](#_Toc210849624)

[3.2. Modelling approaches 7](#_Toc210849625)

[3.3. Sensitivity analysis 8](#_Toc210849626)

# 1. Supplementary figures

**Figure S1.** Flowchart of patient study enrolment and data inclusion/exclusion for the study analysis. Patients were recruited as a convenience sample between 1 April to 30 November 2023 based on review of entries in the hospital’s electronic medical record system, and confirmation of patient eligibility by the treating intensivist. Abbreviations: ICP = intracranial pressure; IOP = intraocular pressure; COVID = coronavirus disease.


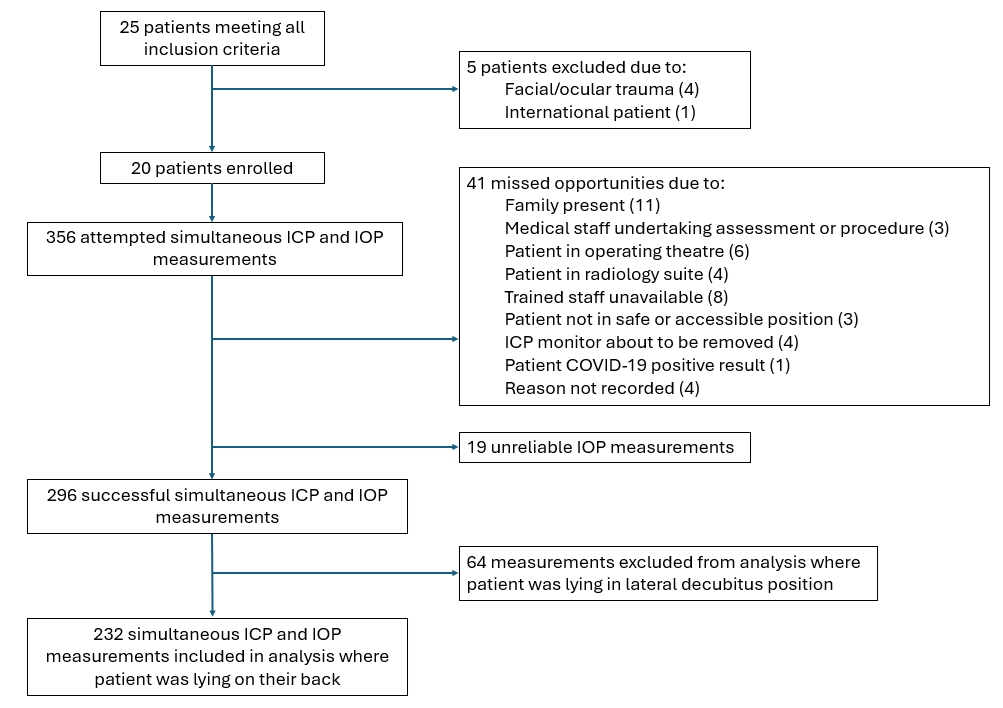


**Figure S2.** Regression diagnostics for the most parsimonious simple linear model for the relationship between ICP and IOP described by ICP = 3.9 + 0.7 × IOP. Scatterplot of residuals versus predicted values showing equal scatter around the value of 0, except for a few points at the highest values of the linear predictor (left) and normality of residuals in the quantile plot (right). Abbreviations: ICP = intracranial pressure; IOP = intraocular pressure


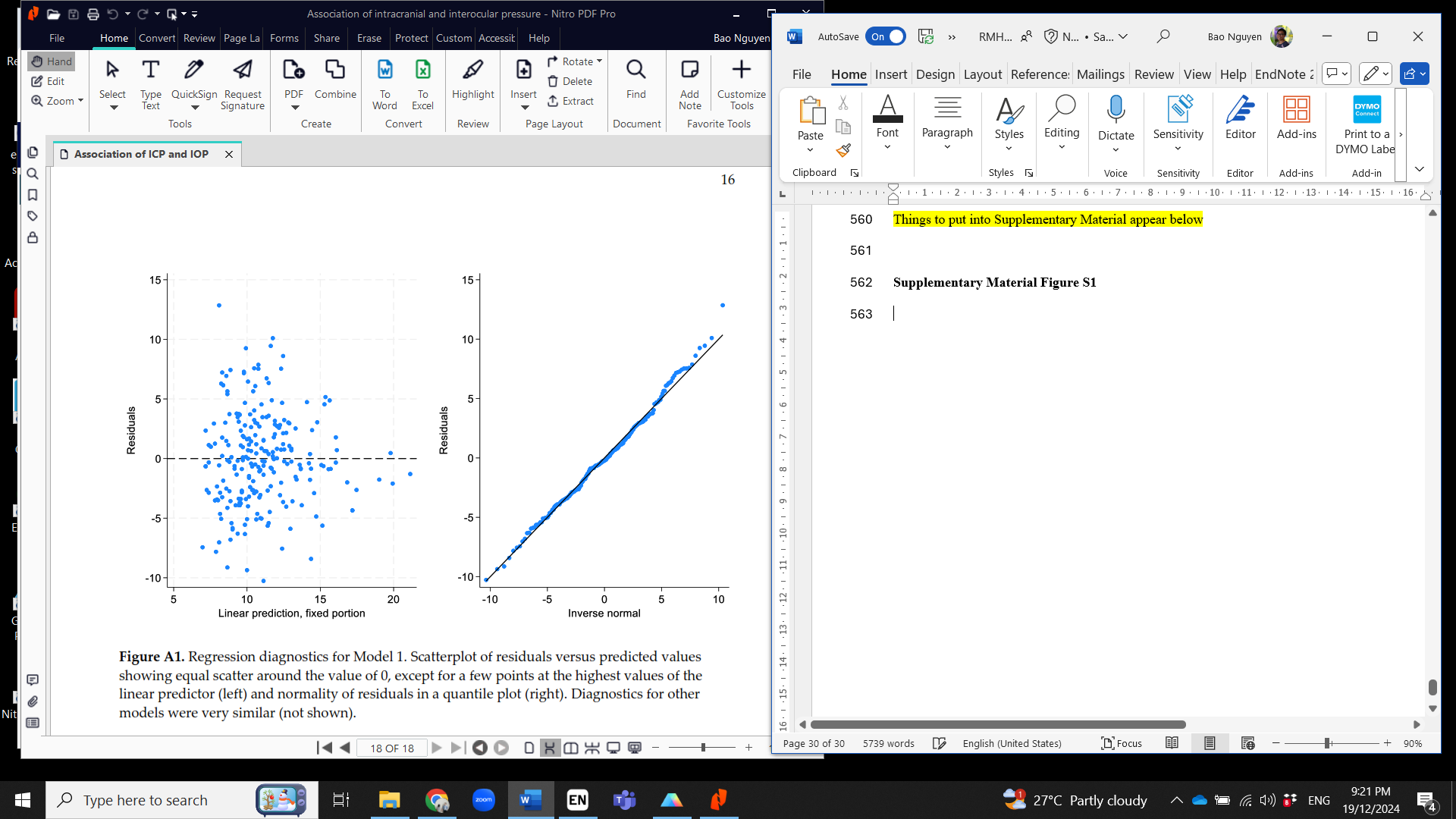


# 2. Supplementary tables

**Table S1.** Study enrolment and measurement characteristics included in the analysis. Abbreviations: ICU = Intensive Care Unit; IOP = intraocular pressure; IQR = interquartile range; ICP = intracranial pressure; SD = standard deviation.

| **Study enrolment characteristic** |  |
| --- | --- |
| Duration (hours) between ICU admission and study enrolment (i.e. first IOP measurement), median [IQR] | 13 [9 – 24] |
| Duration (hours) between ICP monitor insertion and study enrolment (i.e. first IOP measurement), median [IQR] | 14 [11 – 26] |
| Duration (days) of attempted simultaneous ICP and IOP measurements, median [IQR] | 3 [1 – 7] |
| **Measurement characteristic** |  |
| ICP, mmHg, mean ± SD | 12 ± 7 |
| ICP > 20 mmHg, number (%) | 19 (8%) |
| ICP ≤ 20 mmHg, number (%) | 213 (92%) |
| IOP, mmHg, mean ± SD | 10 ± 4 |
| > 21 mmHg, number (%) | 4 (2%) |
| Between 10 and 21 mmHg, number (%) | 100 (43%) |
| < 10 mmHg, number (%) | 128 (55%) |
| Number of successful ICP and IOP measurements per patient, median [IQR] | 9 [6 – 13] |
| Laterality of eye measurement |  |
| Right eye, number (%) | 100 (43%) |
| Left eye, number (%) | 132 (57%) |
| Trained observer taking the IOP measurement |  |
| Observer OG | 139 (60%) |
| Observer BT | 52 (22%) |
| Observer RN | 41 (18%) |
| Head elevation at time of IOP measurement, degrees, mean ± SD | 23 ± 14 |

**Table S2.** Overview of models

| **Model** | **Description** |
| --- | --- |
| 1 | Simple linear association of ICP and IOP |
| 2 | Simple linear association of ICP and IOP allowing the relationship of ICP and IOP to vary for each patient |
| 3 | Simple linear association of ICP and IOP including laterality of eye measurement as a potential source of variation |
| 4 | Simple linear association of ICP and IOP including observer as a potential source of variation |
| 5 | Multivariate linear association of ICP and IOP, adjusting for head elevation |
| 6 | Multivariate linear association of ICP and IOP, adjusting for head elevation and investigating potential effect modification by head elevation |

**Table S3.** Mixed effects linear regression results comparing Model 1 (simple linear association of ICP and IOP) to Model 2 (allowing the relationship of ICP and IOP to vary for each patient), Model 3 (including laterality of eye measurement as a potential source of variation), and Model 4 (including observer as a potential source of variation). Abbreviations: IOP = intraocular pressure; SD = standard deviation; CI = confidence interval.

|  | **Model 1** | **Model 2** | **Model 3** | **Model 4** |
| --- | --- | --- | --- | --- |
|  | Point estimate (95% CI) | Point estimate (95% CI) | Point estimate (95% CI) | Point estimate (95% CI) |
| IOP | 0.70 (0.49, 0.92) | 0.71 (0.42, 1.00) | 0.70 (0.49, 0.92) | 0.7 (0.4, 0.9) |
| Intercept | 3.87 (0.11, 7.63) | 3.84 (-0.83, 8.51) | 3.87 (0.11, 7.63) | 4.6 (0.5, 8.8) |
| SD[Residuals] | 4.11 (3.74, 4.52) | 4.05 (3.67, 4.46) | 4.11 (3.74, 4.52) | 4.0 (3.7, 4.4) |
| SD[Intercept] Patient | 6.49 (4.59, 9.17) | 8.31 (4.24, 16.28) | 6.49 (4.49, 9.17) | 6.5 (4.6, 9.2) |
| SD[IOP] Slope |  | 0.32 (0.10, 1.05) |  |  |
| Correlation[Slope, Intercept] |  | -0.67 (-0.98, 0.61) |  |  |
| SD[Intercept] Laterality |  |  | 0.001 |  |
| SD[Intercept] Observer |  |  |  | 1.36 (0.40, 4.63) |

**Table S4.** Results of mixed effects linear regression comparing separate model fits to the intracranial and intraocular pressure point estimate data, taking into account head elevation as a continuous variable. Abbreviations: IOP = intraocular pressure; SD = standard deviation; CI = confidence interval.

|  | **Model 1** | **Model 5** | **Model 6** |
| --- | --- | --- | --- |
|  | Point estimate (95% CI) | Point estimate (95% CI) | Point estimate (95% CI) |
| IOP | 0.70 (0.49, 0.92) | 0.44 (0.22, 0.66) | 0.39 (0.11, 0.67) |
| Head elevation |  | -0.13 (-0.17, -0.09) | -0.15 (-0.26, -0.05) |
| IOP × Head elevation |  |  | 0.003 (-0.007, 0.013) |
| Intercept | 3.87 (0.11, 7.63) | 9.91 (5.70, 14.10) | 10.41 (5.84, 14.97) |
| SD[Residuals] | 4.11 (3.74, 4.52) | 3.79 (3.45, 4.17) | 3.80 (3.45, 4.18) |
| SD[Intercept] Patient | 6.49 (4.59, 9.17) | 6.67 (4.74, 9.39) | 6.69 (4.75, 9.41) |

**Table S5.** Results of the sensitivity analysis for Models 1, 5 and 6 where ICP values ≤ 0 mmHg were omitted. Abbreviations: ICP = intracranial pressure; IOP = intraocular pressure; SD = standard deviation; CI = confidence interval.

|  | **Model 1** | **Model 5** | **Model 6** |
| --- | --- | --- | --- |
|  | Point estimate (95% CI) | Point estimate (95% CI) | Point estimate (95% CI) |
| IOP | 0.67 (0.44, 0.91) | 0.48 (0.22, 0.74) | 0.49 (0.15, 0.84) |
| Head elevation |  | -0.10 (-0.15, -0.04) | -0.09 (-0.24, 0.06) |
| IOP × Head elevation |  |  | -0.001 (-0.13, 0.01) |
| Intercept | 5.02 (0.89, 9.16) | 9.58 (4.65, 14.51) | 9.44 (3.78, 15.10) |
| SD[Residuals] | 4.08 (3.65, 4.56) | 3.94 (3.53, 4.41) | 3.96 (3.54, 4.43) |
| SD[Intercept] Patient | 6.51 (4.47, 9.50) | 6.65 (4.57, 9.67) | 6.65 (4.57, 9.68) |

# 3. Supplementary methods

## 3.1. Statistical analyses

Missed or unsuccessful IOP measurements were treated as missing data for a given individual and those data excluded from analysis. Descriptive statistics and normality (Kolmogorov-Smirnov test) of data were determined using GraphPad Prism version 10.3.1 for Windows (GraphPad Software, Boston, Massachusetts, USA). To quantify the relationship between ICP and IOP, inferential statistics were performed in Stata Statistical Software (Release 18; StataCorp LLC, College Station, Texas, USA). Mixed effects linear regression using restricted maximum likelihood was used for all models to determine the influence of head elevation, laterality of eye measurement, and interobserver variability. Including a random intercept for patient accounted for repeated measures. Separate models of increasing complexity were fit to the data, exploring potential sources of variation. Point estimates and 95% confidence intervals (CI) were calculated for the fixed effects variables. Standard deviations (SD) were used to estimate within- and between-patient variation.

## 3.2. Modelling approaches

For Model 1 (see Table S3), the SD of the random intercepts was estimated to be 6.5 mmHg, indicating that a mixed model was warranted. Model 2 was therefore fit to relax the assumption that the association between ICP and IOP was the same for all patients (by introducing a random effect for the slope and assuming an unstructured covariance structure relating the intercept to the slope), which did not substantially change the IOP point estimate. Model 3 was then compared to Model 1 to consider laterality of eye measurement (left vs right eye) nested in each patient. Similarly, Model 4 was compared to Model 1 to consider the cross effect of observer (assuming the effect due to observer was systematic for all patients). Table S3 shows that these potential sources of variation did not substantially influence the modelled relationship between ICP and IOP.

To consider the influence of head elevation (a continuous variable), head elevation was initially included in a mixed effects linear model (Model 5) described by ICP = 9.9 + 0.4 × IOP – 0.1 × head elevation. While head elevation explained some of the within-patient variation, the width of the confidence intervals for the residuals of Models 1 and 5 were very similar. When an interaction was included between IOP and head elevation (Model 6), the estimated coefficient of the interaction was close to zero and the 95% confidence interval also spanned zero. Thus, precision of the estimates was unaffected by the addition of head elevation (Table S4).

## 3.3. Sensitivity analysis

All available ICP data was included in the primary analysis to incorporate real-world variation, including four ICP values ≤ 0 mmHg. Sensitivity analysis with these values removed (2% of all measurements) did not substantively change any conclusions when mixed effects linear regression took into account head elevation as a continuous variable (Table S5). Thus, the simplest Model 1, without accounting for head elevation, remained the most parsimonious model describing the overall relationship between ICP and IOP.
